# Hyalinizing trabecular tumor of the thyroid gland and its significant diagnostic issue

**DOI:** 10.1186/s13044-017-0042-5

**Published:** 2017-10-10

**Authors:** Dustin J. Jones, Christopher R. Kieliszak, Sanjay S. Patel, Christopher R. Selinsky

**Affiliations:** 1 Department of Otolaryngology, Head and Neck Surgery, The Detroit Medical Center, Detroit, MI USA; 2 Department of Otolaryngology Head and Neck Surgery, OhioHealth Doctors Hospital, 5100 W Broad Street, Columbus, OH 43228 USA; 3 Department of Pathology and Laboratory Medicine, University of Wisconsin Hospital and Clinics, Madison, WI USA

**Keywords:** Hyalinizing trabecular tumor, Thyroid nodule, Thyroid mass, Cytopathology

## Abstract

**Background:**

Hyalinizing trabecular tumors (HTT) are rare follicular cell-derived tumors of the thyroid gland that are infrequently reported in otolaryngology literature. We present here an interesting case of HTT which provides the basis for review of this entity’s clinical characteristics, criteria useful in making the diagnosis, and any currently available therapeutic modalities.

**Case presentation:**

A 70-year-old Caucasian female underwent a CT scan of her chest and was incidentally found to have a nodule within the right thyroid lobe. Gross examination of the excised thyroid lobe revealed a circumscribed and encapsulated lesion (tan / gritty in texture), confined to the gland. Histologic sections of the lesion revealed a circumscribed neoplasm with a trabecular and organoid architecture associated with abundant dystrophic calcification. Neoplastic cells showed a spindled morphology with clumped chromatin and ample eosinophilic cytoplasm.

**Conclusions:**

Histologically, HTT is a follicular cell-derived tumor composed of neoplastic cells arranged in a trabecular pattern with hyalinization and calcification of extracellular material. Distinguishing features of HTT include minimal cytologic atypia with a low nuclear: cytoplasmic ratio, cellular aggregates around hyalinized material, and nuclei with clumped chromatin and occasional grooves and/or pseudoinclusions. Though debated in the literature, the general consensus is that this tumor is a benign entity. It is our hope that additional clinical research will elicit awareness of these rare tumors.

## Background

Hyalinizing Trabecular Tumors (HTT) are rare follicular cell-derived tumors of the thyroid gland that are infrequently reported in otolaryngology literature. While the incidence of HTT has yet to be confirmed, at one hospital, HTT was reportedly found in 0.44–1.3% of all thyroidectomies [1]. HTT presents most frequently between 21 and 80 years of age (Mean 50.5 y/o) [2, 3]. It has the propensity to affect females at a 6:1 ratio. It has been reported in association with chronic lymphocytic thyroiditis, multinodular goiter and rarely can occur with radiation exposure and familial polyposis [2, 4].

Since Carney et al., first described HTT in 1987, similarities in cellular characteristics, structural integrity, and nuclear composition have created challenges and controversy in distinguishing HTT from Medullary Thyroid Carcinoma (MTC) and Papillary Thyroid Carcinoma (PTC). The diagnostic abstruseness of these tumors has further muddled the terminology due to potentially malignant forms of HTT [4]. Synonyms include: Hyalinizing Trabecular Adenoma, Paraganglioma-Like Adenoma, Hyalinizing Trabecular Neoplasm, Hyalinizing Trabecular Tumor, and Hyalinizing Trabecular Carcinoma [5]. We report an interesting case of HTT which presents an opportunity to describe the clinical characteristics, discuss treatment modalities and bolster clinician awareness of this rare tumor.

## Case presentation

**Fig. 1:**
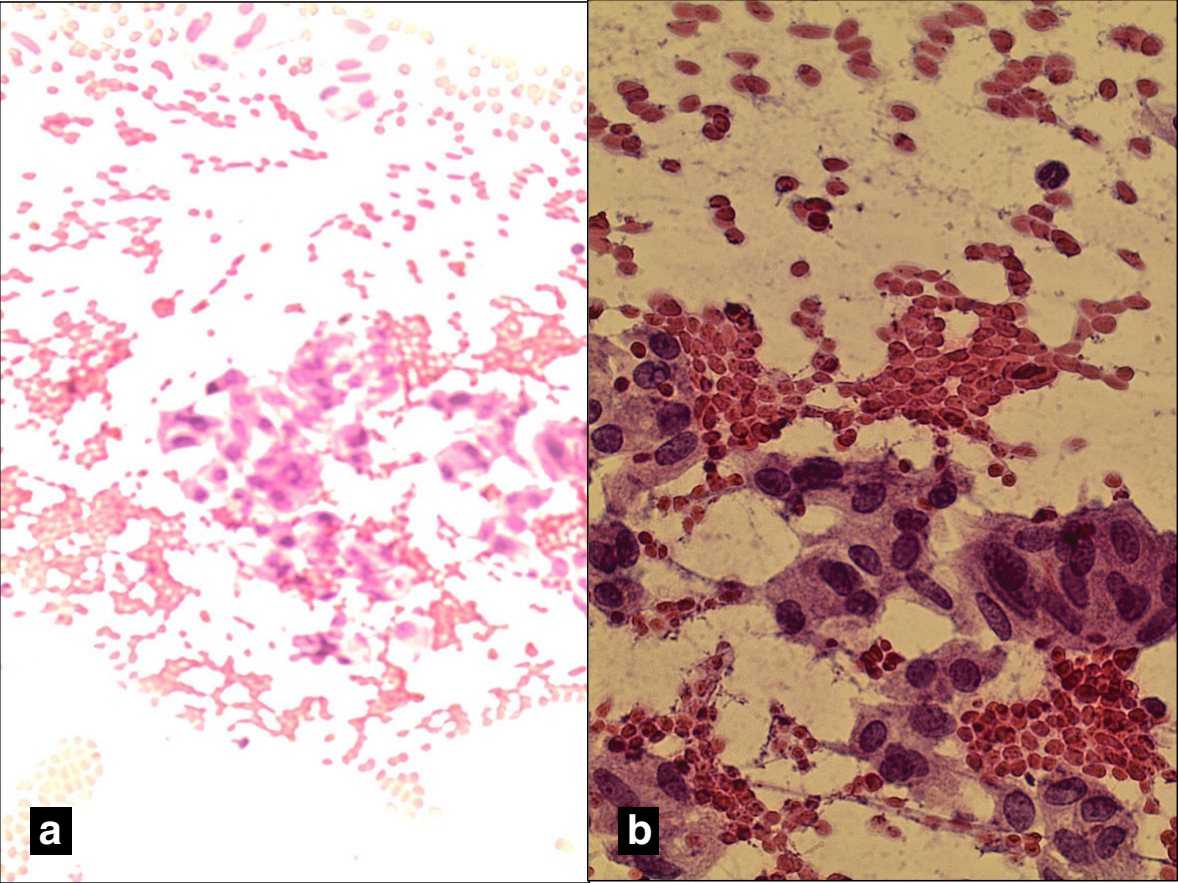
Hematoxylin and Eosin stain of aspirate material from FNA of left thyroid lobe nodule. **a** Cellular aspirate smear consisting of epithelial elements admixed with red blood cells (200X). **b** Follicular cells with elongate/spindle nuclei, dispersed chromatin, and abundant eosinophilic granular cytoplasm (400X)

A 70-year-old Caucasian female underwent a CT scan of her chest and was incidentally found to have a nodule within the right thyroid lobe. A subsequent ultrasound of her thyroid revealed a 1.35 × 1.94 × 1.32 cm mildly heterogeneous and hyperechoic nodule. Cytopathologic examination of aspirate material from an initial ultrasound guided fine needle aspiration (FNA) of the nodule was classified as atypia of Undetermined Significance (AUS) according to Bethesda criteria. A repeat FNA of the right-sided nodule was subsequently determined to be suspicious for malignancy carrying a 60–75% malignancy rate (Fig. 1). She was taken to surgery for hemithyroidectomy and possible total thyroidectomy. Intraoperative frozen section evaluation of the right thyroid lobe revealed papillary thyroid carcinoma and a total thyroidectomy was performed. Gross and microscopic examination of the thyroid tissue with frozen section revealed a right lobe with dimensions of 3.6 × 2.2 × 2.3 cm weighting 7.47 g. Sectioning of the right thyroid lobe identified a 1.3 × 1.1 × 1 cm well- demarcated nodule present within the lobe that was described as a tan with gritty texture. The left thyroid lobe was 2.8 × 2.0 × 1.3 cm weighing 4.57 g, and was described as granular red-purple parenchyma with no defined lesions.

**Fig. 2:**
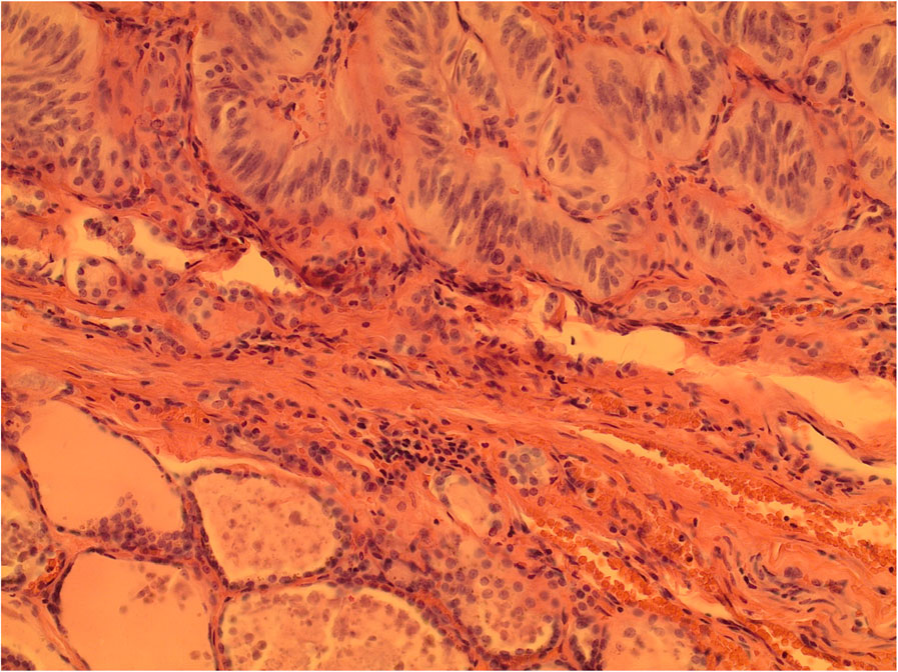
HTT (superior) juxtaposed with uninvolved thyroid parenchyma (inferior) with intervening fibrous capsule (H&E, 200X)

**Fig. 3:**
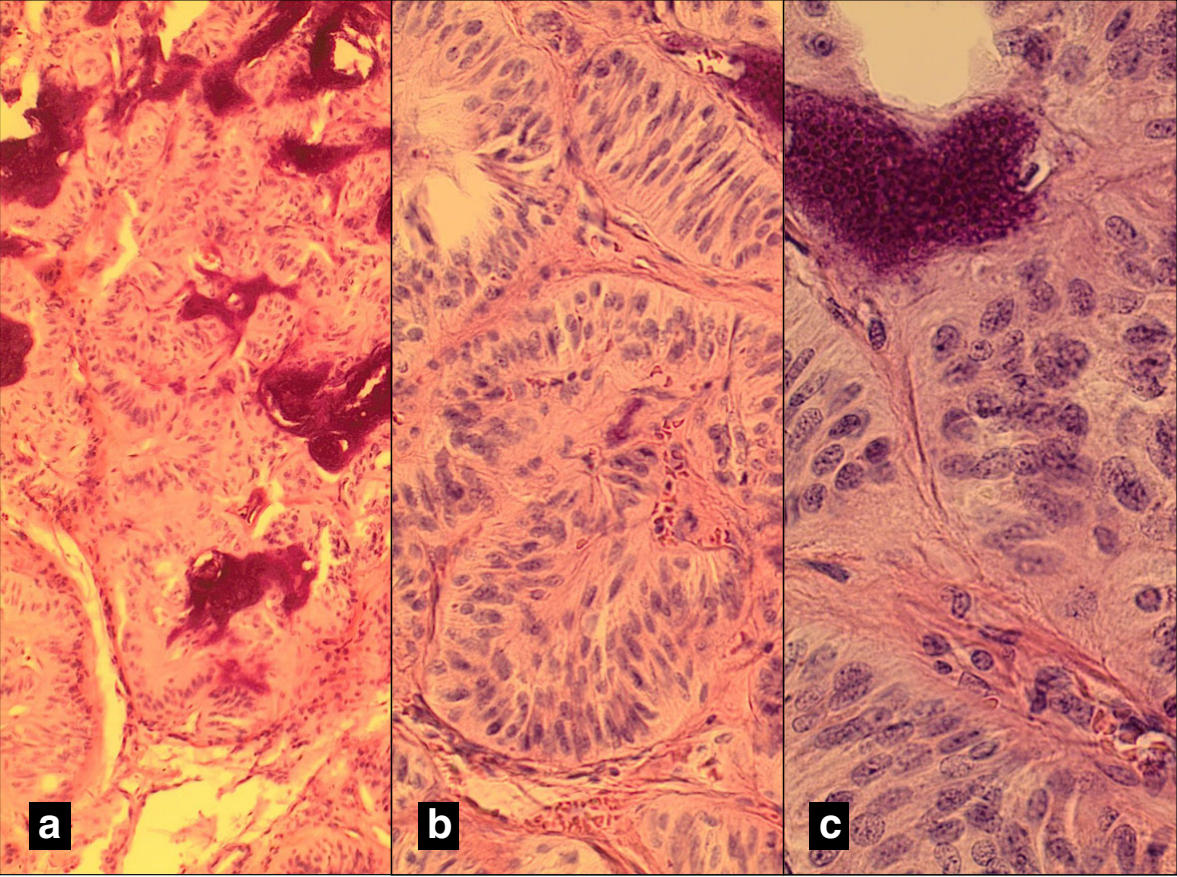
Representative sections of HTT. **a** Organoid and trabecular growth pattern with prominent stromal dystrophic calcifications (H&E, 100X). **b** Higher magnification of features shown in A (H&E, 200X). **c** Neoplastic cells with predominantly spindle morphologic, clumped nuclear chromatin, and abundant granular eosinophilic cytoplasm (H&E, 400X)

Histologic examination of the lesion revealed a trabecular an organoid architecture composed spindle/elongated neoplastic cells with clumped nuclear chromatin, and abundant eosinophilic cytoplasm. Extracellular hyalinization and prominent stromal dystrophic calcification could also be appreciated (Figs. 2 and 3). By immunohistochemical staining, the neoplastic cells were positive for thyroglobulin, vimentin, and weakly reactive for broad-spectrum keratin (AE1/AE3). Chromogranin, calcitonin, cytokeratin 19, and Ki-67 (MIB-1) stains were negative. The cytomorphologic and immunohistochemical features of the neoplasm supported a diagnosis of hyalinizing trabecular tumor (HTT). The diagnosis was made on morphologic and immunohistochemical grounds. Additional ancillary testing in the form of gene mutation or rearrangement studies were not performed.

The patient’s postoperative course was complicated by transient hypoparathyroidism that was addressed with short-term oral calcium and vitamin D supplementation. Additionally, she had postoperative hoarseness. After assessing with video laryngeal stroboscopy, it was determined that she likely had transient right-sided vocal cord paresis due to neuropraxia, as her bilateral recurrent laryngeal nerves were both identified and preserved intraoperatively. Unfortunately, the patient has not followed up in the office since her 1-month post operative visit.

## Discussion and conclusions

Patients presenting with HTT are generally asymptomatic, with size and anatomical location of the tumor influencing clinical symptomatology. As with the vast majority of thyroid nodules the initial workup includes an ultrasound and FNA. Overtly malignant lesions prompt total thyroidectomy, while nodules suspicious for a follicular neoplasm prompt surgical intervention with hemithyroidectomy to obtain specimen to investigate for capsular invasion. Conversely, equivocal cytomorphologic diagnoses (e.g. atypia of undetermined significance [AUS] or follicular lesion of undetermined significance [FLUS]) based on initial FNA necessitate repeat FNA, as risk of malignancy for nodules in these categories ranges between 1 and 15% [6].

The clinical usefulness of frozen section (FS) during intraoperative evaluation remains at great debate and should be directed by FNA findings. When looking at detection rates between benign or malignant nodules, studies have demonstrated similar sensitivity, specificity, and accuracy between FS (87.1%, 100%, 97.8%) and FNA (74.1%, 100%, 95%) [7, 8]. More so, FS utilization following benign FNA findings have shown to increase operative time, cost, and to be of limited usefulness (<1%) in altering surgical decision-making and of no utility following malignant results [8, 9]. Therefore suggesting that the utility of intraoperative FS is most effectively used to parse out FNA findings “suspicious for malignancy” or “AUS/FLUS”. Cetin et al. further supported this by looking at 20 aspirates that were suspicious for malignancy and subsequently found by FS (4 cases) to harbor malignancy [8]. When encountering these situations, FS can alter surgical decisions (up to 20% of cases) that may decrease the need for future completion lobectomy and have aligned with sensitivity (~80%) findings supported by the literature [7–9]. In our case, we encountered a situation where initial FNA revealed AUS and the subsequent aspirate was suspicious for malignancy. Since frozen section revealed papillary thyroid carcinoma, a total thyroidectomy was performed. Intraoperatively, the tumor grossly has a yellow-brown to tan appearance and is well encapsulated, consistent with its benign behavior, although, malignant forms have been reported [10]. In contrast, intraoperative gross evaluations of PTC often reveal grey-white, nonencapsulated, and infiltrative lesions [10].

Histologically, HTT is a follicular cell-derived tumor composed of neoplastic cells arranged in a trabecular pattern with hyalinization and calcification of extracellular material [1, 2, 11]. Nuclear grooves and pseudoinclusions are not uncommon, and the neoplastic cells typically have a low nuclear:cytoplasmic ratio [10]. Resemblances to other follicular cell-derived neoplasms add to the difficulty in distinguishing HTT from its more nefarious mimickers such as MTC, PTC, and Paragangliomas. Overlapping histologic features may include hypercellularity, cellular atypia with cytoplasmic invaginations, psammoma bodies, and nuclear pseudo-inclusions and/or grooves [1, 2, 7, 11]. Distinguishing features of HTT, although subtle, include only minimal cytologic atypia with low nuclear:cytoplasmic ratios, cellular aggregates around hyalinized material, and clumped or fine chromatin rather than optically clear chromatin [10].

In 2012, the notion was proposed that HTT could potentially acquire mutations leading to RET/PTC expression and malignant transformation into PTC [1]. Due to the ambiguity of malignant potential, complete resection, near-total thyroidectomy, or lobectomy are accepted as the current treatment modality [6, 12]. The largest collection of patients (*n* = 119) to our knowledge were collected over a 20-year period and were subsequently diagnosed as a hyalinizing trabecular tumor [2]. The criteria to meet diagnosis was based on histological nuclear and structural (psamomma bodies, pseudoinclusions, frequent grooves, trabecular and organoid architecture) composition [2]. Their findings concluded that only one case showed capsular or vascular invasion that was subsequently found to also have pulmonary metastasis [2]. Carney et al. concluded that the majority of HTT behave as a benign thyroid neoplasm and classification as an “adenoma” would be appropriate [2].

As a consequence of the ambiguity surrounding HTT, up to three quarters (44–71%) of patients are being over treated with total or subtotal thyroidectomy opposed to lobectomy alone [10]. Conversely, it has been proposed that HTT should be conservatively followed with close follow-up or lobectomy instead of upfront total-thyroidectomy [5]. In our case, total thyroidectomy was prompted by PTC in the right thyroid lobe identified by intraoperative frozen section evaluation.

Additionally, Hirokawa et al. established Ki-67 (MIB-1) as a potentially useful marker in establishing a diagnosis of HTT [13]. Furthermore, they investigated similarities in immunohistochemical staining patterns among 17 cases (12 unique cases of HTT; 5 unique cases of PTC) for cytokeratins (CK) (7,14,16,17,18,19,20) and high molecular weight (HMW) cytokeratins (1,5,10,14) in attempt to further differentiate HTT from PTC [14]. This was performed to add support that HTT could be identified as a separate unique entity opposed to a follicular variant of PTC [14]. Their results were significant for near uniform reactivity among the two entities for CK 7, 18; with HTT demonstrating variable reactivity for CK 19 versus PTC showed strong expression. Additionally, while, a limited sample size, HTT showed no reactivity to HMW-CK, whereas, PTC showed strong (90–100% of cells) reactivity in nearly all cases [14]. Their findings suggest that in addition to Ki-67, HMW-CK could serve as a basis for delineating theses two entities when FNA and FS results are equivocal. We present a case that fulfills the architectural diagnostic criteria of trabecular and organoid growth pattern, hyalinized stroma, and spindle cells which are nonreactive by Ki-67 (MIB-1) immunohistochemical stain [2]. While the use of Ki-67, when present, has been identified as a useful marker in delineating between HTT, PTC, and MTC; its absence or non-reactivity does not exclude the diagnosis of HTT [15, 16]. Casey et al. demonstrated reactivity in 71% of FNA aspirates and concluded that reasons for non-reactivity is lacking. Specific explanations for the variability are scarce, however, potential limitations have been proposed such as: (1) expression of Ki-67 in the cell membrane but not in the nuclei of the tumors. (2) Sensitivity in staining of Ki-67 based on Immunohistological (IHC) method utilized. (3) Cell membrane staining may be influenced by antibody (Ki-67) pre-incubation preparation of the specimen and slides. (4) Increased number of ki-67 negative specimens when Diff-Quick methods preceded IHC staining [15, 16]. These findings, given their lack of true specificity, prompt the need for an established diagnostic criterion to more clearly delineate HTT from its follicular cell-derived neoplastic counterparts, specifically, PTC and MTC.

Given the general consensus of HTT being benign, it is our hope that additional clinical research will elicit awareness of these rare tumors. Additional retrospective and prospective examination of HTT of cases may hopefully foster the development of updated diagnostic criteria and treatment protocols that emphasize watchful waiting over aggressive, and potentially unnecessary, surgical intervention.

## Abbreviations

AUS: Atypia of undetermined significance
FLUS: Follicular lesion of undetermined significance
FNA: Fine needle aspiration
HTT: Hylanizing trabecular tumor
MTC: Medullary thyroid carcincoma
PTC: Papillary thyroid carcinoma
